# HiC-Pro: an optimized and flexible pipeline for Hi-C data processing

**DOI:** 10.1186/s13059-015-0831-x

**Published:** 2015-12-01

**Authors:** Nicolas Servant, Nelle Varoquaux, Bryan R. Lajoie, Eric Viara, Chong-Jian Chen, Jean-Philippe Vert, Edith Heard, Job Dekker, Emmanuel Barillot

**Affiliations:** Institut Curie, Paris, France; INSERM, U900, Paris, France; Mines ParisTech, PSL-Research University, CBIO-Centre for Computational Biology, Fontainebleau, France; Program in Systems Biology, Department of Biochemistry and Molecular Pharmacology, University of Massachusetts Medical School, Worcester, MA USA; Sysra, Yerres, France; CNRS UMR3215, Paris, France; INSERM U934, Paris, France; Annoroad Gene Technology Co., Ltd, Beijing, China; Howard Hughes Medical Institute, Program in Systems Biology, Department of Biochemistry and Molecular Pharmacology, University of Massachusetts Medical School, Worcester, MA USA

**Keywords:** Chromosome conformation, Hi-C, Bioinformatics pipeline, Normalization

## Abstract

HiC-Pro is an optimized and flexible pipeline for processing Hi-C data from raw reads to normalized contact maps. HiC-Pro maps reads, detects valid ligation products, performs quality controls and generates intra- and inter-chromosomal contact maps. It includes a fast implementation of the iterative correction method and is based on a memory-efficient data format for Hi-C contact maps. In addition, HiC-Pro can use phased genotype data to build allele-specific contact maps. We applied HiC-Pro to different Hi-C datasets, demonstrating its ability to easily process large data in a reasonable time. Source code and documentation are available at http://github.com/nservant/HiC-Pro.

**Electronic supplementary material:**

The online version of this article (doi:10.1186/s13059-015-0831-x) contains supplementary material, which is available to authorized users.

## Introduction

High-throughput chromosome conformation capture methods are now widely used to map chromatin interactions within regions of interest and across the genome. The use of Hi-C has notably changed our vision of genome organization and its impact on chromatin and gene regulation [1, 2]. The Hi-C technique involves sequencing pairs of interacting DNA fragments, where each mate is associated with one interacting locus. Briefly, cells are crossed-linked, DNA is fragmented using a restriction enzyme [3] or a nuclease [4], and interacting fragments are ligated together. After paired-end sequencing, each pair of reads can be associated to one DNA interaction.

In recent years, the Hi-C technique has demonstrated that the genome is partitioned into domains of different scale and compaction level. The first Hi-C application has described that the genome is partitioned into distinct compartments of open and closed chromatin [3]. Higher throughput and resolution have then suggested the presence of megabase-long and evolutionarily conserved smaller domains. These topologically associating domains are characterized by a high frequency of intra-domain chromatin interactions but infrequent inter-domain chromatin interactions [5, 6]. More recently, very large data sets with deeper sequencing have been used to increase the Hi-C resolution in order to detect loops across the entire genome [7, 8].

As with any genome-wide sequencing data, Hi-C usually requires several millions to billions of paired-end sequencing reads, depending on genome size and on the desired resolution. Managing these data thus requires optimized bioinformatics workflows able to extract the contact frequencies in reasonable computational time and with reasonable resource and storage requirements. The overall strategy to process Hi-C data is converging among recent studies [9], but there remains a lack of stable, flexible and efficient bioinformatics workflows to process such data. Solutions such as the HOMER [10], HICUP [11], HiC-inspector [12], HiCdat [13] and HiCbox [14] pipelines are already available for Hi-C data processing. HOMER offers several functions to analyze Hi-C data but does not perform the mapping of reads nor the correction of systematic biases. HiCdat, HiC-inspector and HiCbox do not allow chimeric reads to be rescued during the mapping of reads. HICUP provides a complete pipeline until the detection of valid interaction products. Using HICUP together with the SNPsplit program [15] allows the extraction of allele-specific interaction products whereas all other solutions do not allow allele-specific analysis. The HiCdat and HiCbox packages offer a means of correcting contact maps for systematic biases. Finally, none of these software were designed to process very large amounts of data in a parallel mode. The hiclib package is currently the most commonly used solution for Hi-C data processing. However, hiclib is a Python library that requires programming skills, such as knowledge of Python and advanced Linux command line, and cannot be used in a single command-line manner. In addition, parallelization is not straightforward and it has limitations with regard to the analysis and normalization of very high-resolution data (Table 1).

**Table 1:** Comparing solutions for Hi-C data processing

	Mapping	Detection of valid interactions	Binning	Correction of systematic noise	Parallel implementation	Allele-specific analysis
HOMER		x	x			
HICUP	x	x				x
HiC-inspector	x^a^	x	x			
HiC-Box	x^a^	x	x	x		
HiCdat	x^a^	x	x	x		
Hiclib	x	x	x	x		
HiC-Pro	x	x	x	x	x	x

HOMER [10] offers several programs to analysis Hi-C data from aligned reads. ^a^HiC-inpector [12], HiCdat [13] and HiC-Box [14] do not allow chimeric reads to be rescued during the mapping. HICUP [11] provides a complete pipeline until the detection of valid interaction products. It can be used together with the SNPsplit software [15] to extract allele-specific mapped reads. The hiclib Python library [17] can be applied for all analysis steps but requires good programming skills and cannot be used in a single command-line manner. None of these software enable very large amounts of data to be processed easily in a parallel mode. Note that HOMER, hiclib and HiCdat also offer additional functions for downstream analysis. In the case of HiC-Pro, the downstream analysis is supported by the HiTC BioConductor package [28]

Here, we present HiC-Pro, an easy-to-use and complete pipeline to process Hi-C data from raw sequencing reads to normalized contact maps. HiC-Pro allows the processing of data from Hi-C protocols based on restriction enzyme or nuclease digestion such as DNase Hi-C [4] or Micro-C [16]. When phased genotypes are available, HiC-Pro is able to distinguish allele-specific interactions and to build both maternal and paternal contact maps. It is optimized and offers a parallel mode for very high-resolution data as well as a fast implementation of the iterative correction method [17].

## Results

### HiC-Pro results and performance

We processed Hi-C data from two public datasets: IMR90 human cell lines from Dixon et al. [6] (IMR90) and from Rao et al. [7] (IMR90_CCL186). The latter is currently one of the biggest datasets available, used to generate up to 5-kb contact maps. For each dataset, we ran HiC-Pro and generated normalized contact maps at 20 kb, 40 kb, 150 kb, 500 kb and 1 Mb resolution. Normalized contact maps at 5 kb were only generated for the IMR90_CCL186 dataset. The datasets were either used in their original form or split into chunks containing 10 or 20 million read pairs.

Using HiC-Pro, the processing of the Dixon’s dataset (397.2 million read pairs split into 84 read chunks) was completed in 2 hours using 168 CPUs (Table 2). Each chunk was mapped on the human genome using four CPUs (two for each mate) and 7 GB of RAM Processing the 84 chunks in parallel allows extraction of the list of valid interactions in less than 30 minutes. All chunks were then merged to generate and normalize the genome-wide contact map.

**Table 2:** HiC-Pro performance and comparison with hiclib

Dataset	IMR90	IMR90	IMR90	IMR90_CCL186
Number of reads	397,200,000	397,200,000	397,200,000	1,535,222,082
Pipeline	hiclib	HiC-Pro	HiC-Pro parallel	HiC-Pro parallel
Number of input files	10	10	84	160
Number of jobs	1	1	42	80
Number of CPUs per job	8	8	4	4
Maximum memory	10	7	7	24
Wall time	28:24	14:32	02:15	11:49
Mapping	22:03	10:31	00:21	05:56
Filtering	00:30	03:10	00:05	00:36
Merge		00:20	00:18	00:50
Contacts maps	01:45	00:15	00:15	00:42
Normalization	04:06	01:16	01:16	03:49

HiC-Pro was run on the IMR90 Hi-C dataset from Dixon et al. and Rao et al. in order to generate contact maps at resolutions of 20 kb, 40 kb, 150 kb, 500 kb and 1 Mb. Contact maps at 5 kb were also generated for the IMR90_CCL186 dataset. The CPU time for each step of the pipeline is reported and compared with the hiclib Python library. The reported results include time of writing contact maps in text format. Times are minutes:seconds

In order to compare our results with the hiclib library, we ran HiC-Pro on the same dataset, and without initial read splitting, using eight CPUs. HiC-Pro performed the complete analysis in less than 15 hours compared with 28 hours for the hiclib pipeline. The main difference in speed is explained by our two-step mapping strategy compared with the iterative mapping strategy of hiclib, which aligned the 35 base pair (bp) reads in four steps. Optimization of the binning process and implementation of the normalization algorithm led to a three-fold decrease in time to generate and normalize the genome-wide contact map.

The IMR90 sample from the Rao dataset (1.5 billion read pairs split into 160 read chunks) was processed in parallel using 320 CPUs to generate up to 5-kb contact maps in 12 hours, demonstrating the ability of HiC-Pro to analyze very large amounts of data in a reasonable time. At a 5-kb resolution, we observe the presence of chromatin loops as described by Rao et al. [7] (Figure S1 in Additional file 1). The merged list of valid interactions was generated in less than 7.5 hours. Normalization of the genome-wide contact map at 1 Mb, 500 kb, 150 kb, 40 kb, 20 kb and 5 kb was performed in less than 4 hours. Details about the results and the implementation of the different solutions are available in Additional file 1.

**Fig. 1:**
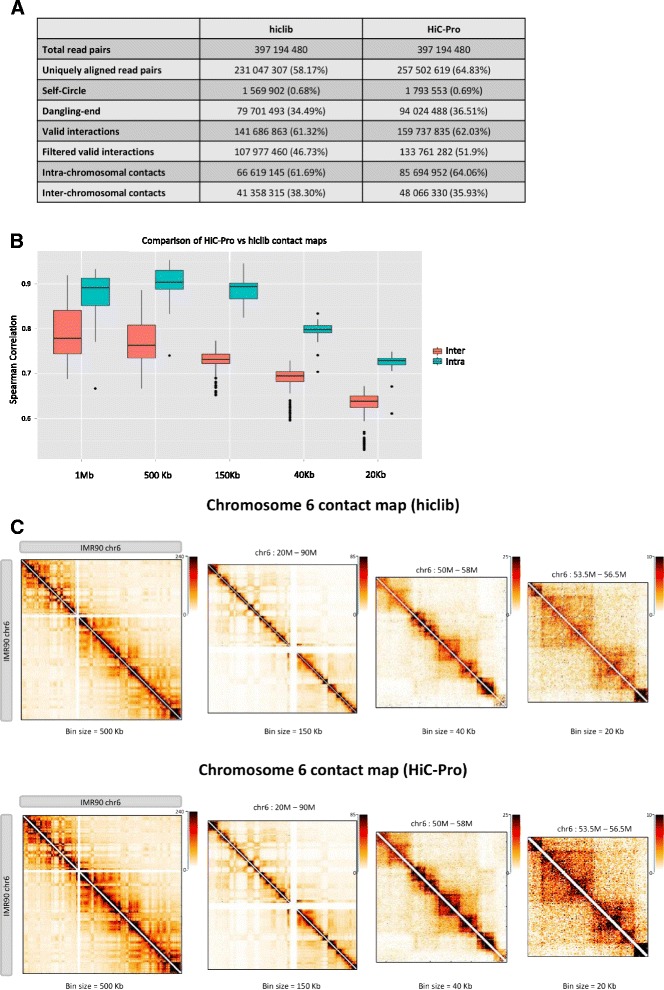
Comparison of HiC-Pro and hiclib processing. **a** Both pipelines generate concordant results across processing steps. The fraction of uniquely aligned read pairs is calculated on the total number of initial reads. Self-circle and dangling-end fractions are calculated on the total number of aligned read pairs. Intra- and inter-chromosomal contacts are calculated as a fraction of filtered valid interactions. **b** Boxplots of the Spearman correlation coefficients of intra- and inter-chromosomal maps generated at different resolutions by both pipelines. **c** Chromosome 6 contact maps generated by hiclib (*top*) and HiC-Pro (*bottom*) at different resolutions. The chromatin interaction data generated by the two pipelines are highly similar

Finally, we compared the Hi-C processing results of hiclib and HiC-Pro on the IMR90 dataset. Although the processing and filtering steps of the two pipelines are not exactly the same, we observed a good concordance in the results (Fig. 1). Using default parameters, HiC-Pro is less stringent than hiclib and used more valid interactions to build the contact maps. The two sets of normalized contact maps generated at different resolutions are highly similar (Fig. 1c). We further explored the similarity between the maps generated by the two pipelines by computing the Spearman correlation of the normalized intra-chromosomal maps. The average correlation coefficient across all chromosomes at different resolutions was 0.83 (0.65–0.95). Finally, since the inter-chromosomal data are usually very sparse, we summarized the inter-chromosomal signal using two one-dimensional coverage vectors of rows and columns [18, 19]. The average Spearman correlation coefficient of all coverage vectors between hiclib and HiC-Pro inter-chromosomal contact maps was 0.75 (0.46–0.98).

### Implementation of the iterative correction algorithm

We provide an implementation of the iterative correction procedure which emphasizes ease of use, performance, memory-efficiency and maintainability. We obtain higher or similar performance on a single core compared with the original ICE implementation from the hiclib library (Table 2) and from the HiCorrector package [20] (Table 3).

**Table 3:** Performance of iterative correction on IMR90 data

	HiC-Pro – Iced (dense – 1 CPU)	HiC-Pro – Iced (sparse – 1 CPU)	HiCorrector – MES (dense – 1 CPU)	HiCorrector – MEP (dense – 8 CPUs)
IMR90 1Mbp	00:00:12	00:00:25	00:00:25	00:00:06
IMR90 500 kbp	00:00:40	00:01:30	00:02:15	00:00:22
IMR90 150 kbp	-	00:04:28	00:13:21	00:03:10
IMR90 40 kbp	-	00:07:19	02:35:34	00:35:43
IMR90 2 0kbp	-	00:08:36	12:57:17	02:34:05

HiC-Pro is based on a fast implementation of the iterative correction algorithm. We therefore compare our method with the MES (Memory-Efficient Sequential) and MEP (Memory-Efficient Parallel) algorithms of the HiCorrector software [20] for Hi-C data normalization (hours:minutes:seconds). All algorithms were terminated after 20 iterations (see Additional file 1 for details)

The HiCorrector package provides a parallel version of the iterative correction for dense matrices. We therefore compared the performance of HiCorrector with the HiC-Pro normalization at different Hi-C resolutions (Table 3). All algorithms were terminated after 20 iterations for the purpose of performance comparison, as each iteration requires nearly the same running time. Choosing dense or sparse matrix-based implementation is dependent on the Hi-C data resolution and on the depth of coverage. Although our implementation can be run in either sparse or dense mode, the available data published at resolutions of 5–40 kb are currently characterized by a high degree of sparsity. At each level of Hi-C contact map resolution, we compared our dense or sparse implementation with the parallel and/or sequential version of HiCorrector. Our results demonstrate that using a compressed sparse row matrix structure is more efficient on high resolution contact maps (<40 kb) than using parallel computing on dense matrices. As expected for low resolution contact maps (1 Mb, 500 kb), using a dense matrix implementation is more efficient in time, although the gain, in practice, remains negligible.

The code for the normalization is available as a standalone package (https://github.com/hiclib/iced) as well as being included in HiC-Pro. Our implementation based on sparse row matrices is able to normalize a 20-kb human genome map in less than 30 minutes with 5 GB of RAM (Table 3). Genome-wide normalization at 5 kb can be achieved in less than 2.5 hours with 24 GB of RAM. Thus, compared to existing solutions, our implementation substantially speeds up and facilitates the normalization of Hi-C data prior to downstream analysis.

### Allele-specific contact maps

We used HiC-Pro to generate allele-specific contact maps for the human GM12878 cell line. Differences in paternal and maternal X chromosome organization were recently described, with the presence of mega-domains on the inactive X chromosome, which are not seen in the active X chromosome [7, 21, 22]. We used HiC-Pro to generate the maternal and paternal chromosome X contact maps of the GM12878 cell line using the Hi-C dataset published by Selvaraj et al. [23]. Phasing data were gathered from the Illumina Platinum Genomes Project [24]. Only good quality heterozygous phased single-nucleotide polymorphisms (SNPs) were selected. The final list contained 2,239,492 SNPs. We then masked the human genome hg19 by replacing the SNP position by an ‘N’ using the BEDTools utilities [25] and generated the new bowtie2 indexes. In practice, the allele-specific analysis can be easily performed by simply specifying to HiC-Pro the list of SNPs and the N-masked indexes for read alignment through the configuration file.

**Fig. 2:**
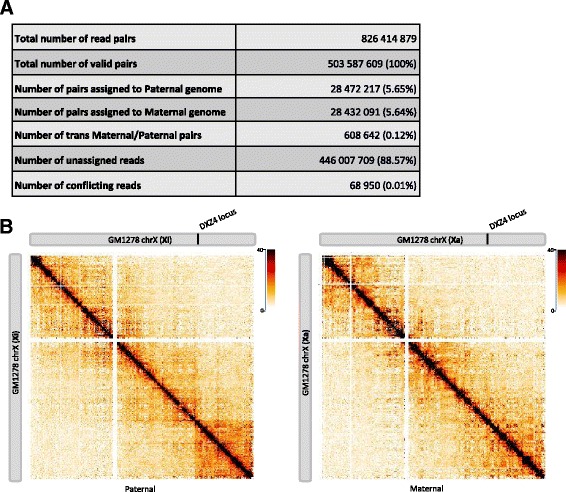
Allele-specific analysis. **a** Allele-specific analysis of the GM12878 cell line. Phasing data were gathered from the Illumina Platinum Genomes Project. In total, 2,239,492 high quality SNPs from GM12878 data were used to distinguish both alleles. Around 6 % of the read pairs were assigned to each parental allele and used to build the allele-specific contact maps. **b** Intra-chromosomal contact maps of inactive and active X chromosome of the GM12878 cell line at 500-kb resolution. The inactive copy of chromosome X is partitioned into two mega-domains which are not seen in the active X chromosome. The boundary between the two mega-domains lies near the DXZ4 micro-satellite

Among the initial 826 million read pairs, 61 % were classified as valid interactions by HiC-Pro. Around 6 % of valid interactions were then assigned to either the paternal or maternal genome and used to construct the haploid maps. As expected, the inactive X chromosome map is partitioned into two mega-domains (Fig. 2). The boundary between the two mega-domains lies near the DXZ4 micro-satellite.

## Materials and methods

### HiC-Pro workflow

**Fig. 3:**
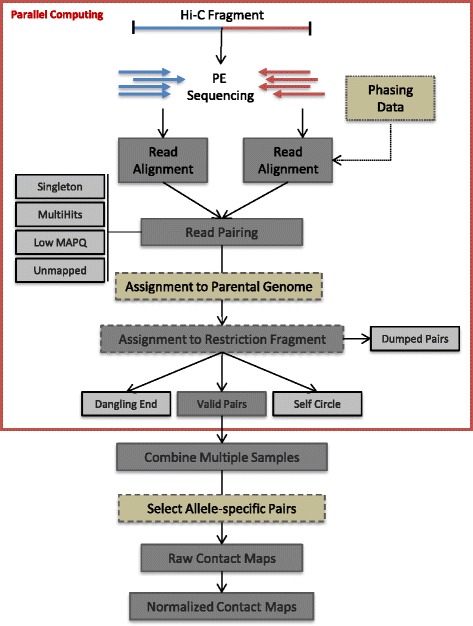
HiC-Pro workflow. Reads are first aligned on the reference genome. Only uniquely aligned reads are kept and assigned to a restriction fragment. Interactions are then classified and invalid pairs are discarded. If phased genotyping data and N-masked genome are provided, HiC-Pro will align the reads and assign them to a parental genome. For the Hi-C protocol based on restriction enzyme digestion, the read pairs will then be assigned to a restriction fragment and invalid ligation products will be filtered out. These first steps can be performed in parallel for each read chunk. Data from multiple chunks are then merged and binned to generate a single genome-wide interaction map. For allele-specific analysis, only pairs with at least one allele-specific read are used to build the contact maps. The normalization is finally applied to remove Hi-C systematic bias on the genome-wide contact map. *MAPQ* Mapping Quality , *PE* paired end

HiC-Pro is organized into four distinct modules following the main steps of Hi-C data analysis: (i) read alignment, (ii) detection and filtering of valid interaction products, (iii) binning and (iv) contact map normalization (Fig. 3).

#### Mapping

**Fig. 4:**
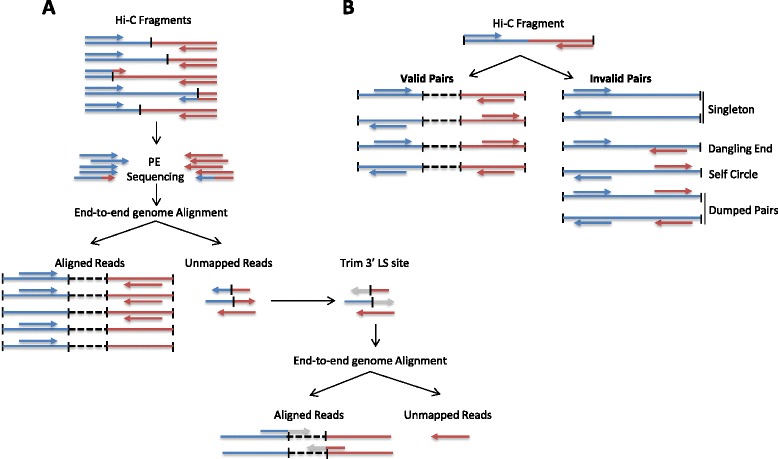
Read pair alignment and filtering. **a** Read pairs are first independently aligned to the reference genome using an end-to-end algorithm. Then, reads spanning the ligation junction which were not aligned in the first step are trimmed at the ligation site and their 5′ extremity is realigned on the genome. All aligned reads after these two steps are used for further analysis. **b** According to the Hi-C protocol, digested fragments are ligated together to generate Hi-C products. A valid Hi-C product is expected to involve two different restriction fragments. Read pairs aligned on the same restriction fragment are classified as dangling end or self-circle products, and are not used to generate the contact maps. *PE* paired end, *LS Ligation Site *

Read pairs are first independently aligned on the reference genome to avoid any constraint on the proximity between the two reads. Most read pairs are expected to be uniquely aligned on the reference genome. A few percent, however, are likely to be chimeric reads, meaning that at least one read spans the ligation junction and therefore both interacting loci. As an alternative to the iterative mapping strategy proposed by Imakaev et al. [17], we propose a two-step approach to rescue and align those reads (Fig. 4a). Reads are first aligned on the reference genome using the bowtie2 end-to-end algorithm [26]. At this point, unmapped reads are mainly composed of chimeric fragments spanning the ligation junction. According to the Hi-C protocol and the fill-in strategy, HiC-Pro is then able to detect the ligation site using an exact matching procedure and to align back on the genome the 5′ fraction of the read. Both mapping steps are then merged in a single alignment file. Low mapping quality reads, multiple hits and singletons can be discarded.

#### Detection of valid interactions

Each aligned read can be assigned to one restriction fragment according to the reference genome and the selected restriction enzyme. Both reads are expected to map near a restriction site, and with a distance within the range of molecule size distribution after shearing. Fragments with a size outside the expected range can be discarded if specified but are usually the result of random breaks or star activity of the enzyme, and can therefore be included in downstream analysis [17]. Read pairs from invalid ligation products, such as dangling end and self-circle ligation, are discarded (Fig. 4b). Only valid pairs involving two different restriction fragments are used to build the contact maps. Duplicated valid pairs due to PCR artifacts can also be filtered out. Each read is finally tagged in a BAM file according to its mapping and fragment properties (Figure S2 in Additional file 1). In the context of Hi-C methods which are not based on restriction enzyme digestion, no filtering of restriction fragments is applied. The uniquely mapped read pairs are directly used to build the contact maps. However, one way to filter out artifacts such as self-ligation is to discard intra-chromosomal pairs below a given distance threshold [4]. HiC-Pro therefore allows these short range contacts to be filtered out.

#### Binning

In order to generate the contact maps, the genome is divided into bins of equal size, and the number of contacts observed between each pair of bins is reported. A single genome-wide interaction map containing both raw intra- and inter-chromosomal maps is generated for a set of resolutions defined by the user in the configuration file.

#### Normalization

In theory, the raw contact counts are expected to be proportional to the true contact frequency between two loci. As for any sequencing experiment, however, it is known that Hi-C data contain different biases mainly due to GC content, mappability and effective fragment length [18, 19]. An appropriate normalization method is therefore mandatory to correct for these biases. Over the last few years, several methods have been proposed using either an explicit-factor model for bias correction [19] or implicit matrix balancing algorithm [17, 27]. Among the matrix balancing algorithm, the iterative correction of biases based on the Sinkhorn-Knopp algorithm has been widely used by recent studies due to its conceptual simplicity, parameter-free nature and ability to correct for unknown biases, although its assumption of equal visibility across all loci may require further exploration. In theory, a genome-wide interaction matrix is of size O(N^2^), where N is the number of genomic bins. Therefore, applying a balancing algorithm on such a matrix can be difficult in practice, as it requires a significant amount of memory and computational time. The degree of sparsity of the Hi-C data is dependent on the bin size and on the sequencing depth of coverage. Even for extremely large sequencing coverage, the interaction frequency between intra-chromosomal loci is expected to decrease as the genomic distance between them increases. High-resolution data are therefore usually associated with a high level of sparsity. Exploiting matrix sparsity in the implementation can improve the performance of the balancing algorithm for high-resolution data. HiC-Pro proposes a fast sparse-based implementation of the iterative correction method [17], allowing normalization of genome-wide high-resolution contact matrices in a short time and with reasonable memory requirements.

### Quality controls

**Fig. 5:**
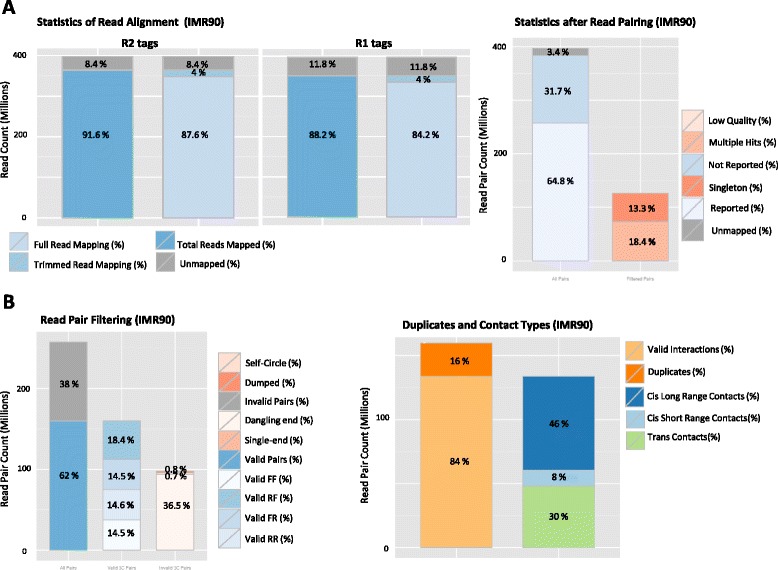
HiC-Pro quality controls. Quality controls reported by HiC-Pro (IMR90, Dixon et al. data). **a** Quality control on read alignment and pairing. Low quality alignment, singleton and multiple hits are usually removed at this step. **b** Read pair filtering. Read pairs are assigned to a restriction fragment. Invalid pairs, such as dangling-end and self-circle, are good indicators of the library quality and are tracked but discarded for subsequent further analysis. The fractions of duplicated reads, as well as short range versus long range interactions, are also reported

To assess the quality of a Hi-C experiment, HiC-Pro performs a variety of quality controls at different steps of the pipeline (Fig. 5). The alignment statistics are the first available quality metric. According to the reference genome, a high-quality Hi-C experiment is usually associated with a high mapping rate. The number of reads aligned in the second mapping step is also an interesting control as it reflects the proportion of reads spanning the ligation junction. An abnormal level of chimeric reads can reflect a ligation issue during library preparation. Once the reads are aligned on the genome, the fraction of singleton or multiple hits is usually expected to be low. The ligation efficiency can also be assessed using the filtering of valid and invalid pairs. As ligation is a random process, it is expected that 25 % of each valid ligation class will be defined by distinct read pair orientation. In the same way, a high level of dangling-end or self-circle read pairs is associated with a bad quality experiment, and reveals a problem during the digestion, fill-in or ligation steps.

Additional quality controls, such as fragment size distribution, can be extracted from the list of valid interaction products (Figure S3 in Additional file 1). A high level of duplication indicates poor molecular complexity and a potential PCR bias. Finally, an important metric is the fraction of intra- and inter-chromosomal interactions, as well as long-range versus short-range intra-chromosomal interactions. As two genomic loci close on the linear genome are more likely to randomly interact, a strong diagonal is expected on the raw contact maps. A low quality experiment will result in a low fraction of intra-chromosomal interactions depending on the organism and the biological context. A high quality Hi-C experiment on the human genome is typically characterized by at least 40 % of intra-chromosomal interactions [9]. In the same way, a high quality experiment is usually characterized by a significant fraction (>40 %) of long-range intra-chromosomal valid pairs [7].

### Speed and scalability

Generating genome-wide contact maps at 40 to 1 kb resolution requires a sequencing depth of hundreds of millions to multi-billions of paired-end reads depending on the organism [7, 8]. However, the main processing steps from read mapping to fragment reconstruction can be optimized using parallel computation of read chunks, significantly reducing the time taken by the Hi-C data processing. Next, all valid interactions are merged to remove the duplicates and to generate the final contact maps.

The user can easily run the complete analysis workflow with a single command line either on a single laptop or on a computer cluster. Analysis parameters are all defined in a single configuration file. In addition, HiC-Pro is modular and sequential, allowing the user to focus on a sub-part of the processing without running the complete workflow. In this way, HiC-Pro can also be used to complement other methods, for instance, by running the workflow from already aligned files, or by simply normalizing published raw contact maps.

The main steps of the pipeline are implemented in Python and C++ programming languages and are based on efficient data structures, such as compressed sparse row matrices for contact count data. Using an adequate data structure allows the data processing to be sped up as well circumvents memory limitations. In this way, HiC-Pro allows a genome-wide iterative correction to be run at very high resolution and in a short time. Our normalization implementation exploits *numpy*’s dense array format and fast operations, *scipy*’s sparse matrices representation and *Cython* to combine C and Python to reach the performance of C executables with the ease of use and maintainability of the Python language.

### Contact map storage

Genome-wide contact maps are generated for resolutions defined by the user. A contact map is defined as a matrix of contact counts and a description of the associated genomic bins and is usually stored as a matrix, divided into bins of equal size. The bin size represents the resolution at which the data will be analyzed. For instance, a human 20 kb genome-wide map is represented by a square matrix of 150,000 rows and columns, which can be difficult to manage in practice. To address this issue, we propose a standard contact map format based on two main observations. Contact maps at high resolution are (i) usually sparse and (ii) expected to be symmetric. Storing the non-null contacts from half of the matrix is therefore enough to summarize all the contact frequencies. Using this format leads to a 10–150-fold reduction in disk space use compared with the dense format (Table 4).

**Table 4:** Comparison of contact map formats

	Dense format (MB)	Sparse symmetric format (MB)
IMR90_CCL186 1 Mbp	27	49
IMR90_CL186 500 kbp	82	181
IMR90_CCL186 150 kbp	822	911
IMR90_CCL186 40 kbp	12,000	1900
IMR90_CL186 20 kbp	45,000	2600
IMR90_CL186 5 kbp	720,000	4200

Disk space for IMR90_CCL186 genome-wide contact maps generated using either the classical dense format or the sparse symmetric format at different resolutions

### Allele-specific analysis

HiC-Pro is able to incorporate phased haplotype information in the Hi-C data processing in order to generate allele-specific contact maps (Fig. 2). In this context, the sequencing reads are first aligned on a reference genome for which all polymorphic sites were first N-masked. This masking strategy avoids systematic bias toward the reference allele, compared with the standard procedure where reads are mapped on an unmasked genome. Once aligned, HiC-Pro browses all reads spanning a polymorphic site, locates the nucleotide at the appropriate position, and assigns the read to either the maternal or paternal allele. Reads without SNP information as well as reads with conflicting allele assignment or unexpected alleles at polymorphic sites are flagged as unassigned. A BAM file with an allele-specific tag for each read is generated and can be used for further analysis. Then, we classify as allele-specific all pairs for which both reads are assigned to the same parental allele or for which one read is assigned to one parental allele and the other is unassigned. These allele-specific read pairs are then used to generate a genome-wide contact map for each parental genome. Finally, the two allele-specific genome-wide contact maps are independently normalized using the iterative correction algorithm.

### Software requirements

The following additional software and libraries are required: the bowtie2 mapper [26], R and the BioConductor packages *RColorBrewer*, *ggplot2*, *grid*, Samtools (>0.1.19), Python (>2.7) with the *pysam*, *bx.python*, *numpy* and *scipy* libraries, and the g++ compiler. Note that a bowtie2 version > 2.2.2 is strongly recommended for allele-specific analysis, because, since this version, read alignment on an N-masked genome has been highly improved. Most of the installation steps are fully automatic using a simple command line. The bowtie2 and Samtools software are automatically downloaded and installed if not detected on the system. The HiC-Pro pipeline can be installed on a Linux/UNIX-like operating system.

## Conclusions

As the Hi-C technique is maturing, it is now important to develop bioinformatics solutions which can be shared and used for any project. HiC-Pro is a flexible and efficient pipeline for Hi-C data processing. It is freely available under the BSD licence as a collaborative project at https://github.com/nservant/HiC-Pro. It is optimized to address the challenge of processing high-resolution data and provides an efficient format for contact map sharing. In addition, for ease of use, HiC-Pro performs quality controls and can process Hi-C data from the raw sequencing reads to the normalized and ready-to-use genome-wide contact maps. HiC-Pro can process data generated from protocols based on restriction enzyme or nuclease digestion. The intra- and inter-chromosomal contact maps generated by HiC-Pro are highly similar to the ones generated by the hiclib package. In addition, when phased genotyping data are available, HiC-Pro allows the easy generation of allele-specific maps for homologous chromosomes. Finally, HiC-Pro includes an optimized version of the iterative correction algorithm, which substantially speeds up and facilitates the normalization of Hi-C data. The code is also available as a standalone package (https://github.com/hiclib/iced).

A complete online manual is available at http://nservant.github.io/HiC-Pro. The raw and normalized contact maps are compatible with the HiTC Bioconductor package [28], and can therefore be loaded in the R environment for visualization and further analysis.

## Additional file

Additional file 1:
**The supplementary data file contains a description of the dataset used for this study as well as details about how the HiC-Pro, hiclib and HiCorrector software were used in practice.** It also includes supplementary figures about the HiC-Pro results and output. (DOCX 1452 kb)

## Abbreviations

PCR: polymerase chain reaction
SNP: single-nucleotide polymorphism
