# Supplementary material for: HiC-Pro: an optimized and flexible pipeline for Hi-C data processing
Source: Genome Biol. 2015 Dec 1;16:259. doi: 10.1186/s13059-015-0831-x (PMC4665391; doi:10.1186/s13059-015-0831-x)
Supplement: Additional file 1: — The supplementary data file contains a description of the dataset used for this study as well as details about how the HiC-Pro, hiclib and HiCorrector software were used in practice. It also includes supplementary figures about the HiC-Pro results and output. (DOCX 1452 kb) [file 13059_2015_831_MOESM1_ESM.docx]

**HiC-Pro: An optimized and flexible pipeline for Hi-C data processing.**

N. Servant, N. Varoquaux, B. R. Lajoie, E. Viara, CJ. Chen, JP. Vert, E. Heard, J. Dekker, E. Barillot

**SUPPLEMENTARY MATERIAL**

1. **Public dataset used.**

We applied the HiC-Pro pipeline on three public dataset available on GEO.

The IMR90 Hi-C contact maps were first published by Dixon et al. at a resolution of 20Kb and 40Kb. The five run of IMR90 replicate 1 (GSM862724) were used and merged, for a total number of 397.2 million read pairs. We refer to this sample in the manuscript as IMR90.

More recently, Rao et al. generate genome-wide contact maps at a resolution of 1-5kb (GSE63525) for nine different cell lines. For the purpose of this paper, we applied HiC-Pro on the IMR90 cell line (GSM1551599, GSM1551600, GSM1551601, GSM1551602, GSM1551603, GSM1551604, GSM1551605). The combined samples represent a sequencing depth of 1.5 billion reads. We refer to this sample in the manuscript as IMR90_CCL186.

The allele specific analysis was performed using the human GM12878 Hi-C data published by Selveraj et al. (GSE48592). Phasing data were gathered from the Illumina Platinum Project v8.0.1 (<http://www.illumina.com/platinumgenomes/>).

1. **Results and implementation**

All pipelines and software were run on the high-performance computing resource of the Institut Curie. Each node has a total of 32 or 48 processors (Intel Xeon 2.2 GHz) and 128 GB memory.

The HiC-Pro version 2.6.0 was used and the *hiclib* library was downloaded from <http://mirnylab.bitbucket.org/hiclib/>. In order to compare the performance between both solutions, we run the pipeline described in the *hiclib*’s repository (hiclib/[examples](https://bitbucket.org/mirnylab/hiclib/src/460c3fbc0f72/examples/?at=default)/pipeline2014/), on a single node with 8 CPUs. Following the *hiclib*'s help pages, the *binnedData* and *highResBinnedData* classes were respectively used for low (>100kb) and high resolution data (<100kb) as illustrated in the *testHighResHiC.py* script.

The HiC-Pro pipeline was run either in normal or parallel mode. HiC-Pro and *hiclib* were compared until the generation of genome-wide normalized contact maps at a resolution of 1Mb, 500Kb, 150Kb, 40Kb and 20Kb. Both pipelines were run with default parameters. The running time includes the export of contact maps in text format.

In order to compare the results generated by both pipelines, we calculated the Spearman correlation coefficient between HiC-Pro and hiclib intra and inter-chromosomal maps at different resolutions. By default hiclib is removing the matrix diagonal before normalizing the data. We therefore apply the same filter on the HiC-Pro contact maps to compare the results. The Spearman correlation coefficients were calculated between all intra-chromosomal maps. Since the inter-chromosomal contact maps are sparse, instead of measuring the correlation directly between the two maps, we computed the Spearman correlation of the one-dimensional coverage vectors of inter-chromosomal maps as proposed by Yaffe and Tanay (2011), and Hu et al. (2012). The results are available in Figure S4.

The HiCorrector package (version 1.1) was downloaded and compiled using openmpi-1.4.5.

We compared the performance of the iterative correction algorithm included in HiC-Pro with HiCorrector on the Dixon et al. IMR90 dataset. We first split the dense matrix files using the *split_data_parallel* tool and the following command line; “*mpirun -np 8 split_data_paralllel DENSE_MATRIX_FILE NB_ROWS ./ 8 1024 job_id*” where *DENSE_MATRIX_FILE* is the path to the dense matrix and *NB_ROWS* the number of matrix rows. The genome wide contact maps were therefore split into 7 sub-matrices for 1M, 500Kb, 150Kb resolutions, 28 sub-matrices for the 40Kb resolution and 91 for the 20 Kb resolution.

The iterative correction was then applied using the ICE-MES and ICE-MEP methods on the genome-wide contact map. All algorithms were terminated after 20 iterations.

We ran the ICE_MEP method using the following parameters; “*mpirun -np 8 ic_mep –useSplitInputFiles –numRows=NB_RAWS –maxIteration=20 –numTask=8*

*–memSizePerTask=1024 –jobID=job_id*”. The ICE_MES method was run using the following parameters; “”*ic_mes DENSE_MATRIX_FILE 5000 3115 20 0 0*”.

The HiC-Pro normalization (1 CPU) was run using the ice script and the following parameters; “*--max_iter 20 –eps 1e-15 –filtering_perc 0*”. The “*--dense*” option was added for the dense matrices. All input and output files were stored in the local *scratch* folder to limit the I/O time due to NFS system.

**SUPPLEMENTARY FIGURES.**

chr3: 63 .1Mb – 64.7Mb

chr16 : 85.7Mb – 87.4Mb

chr21 : 28Mb – 30.3Mb

Bin size = 5 Kb

chr16

chr21

chr3

chr3

chr16

chr21


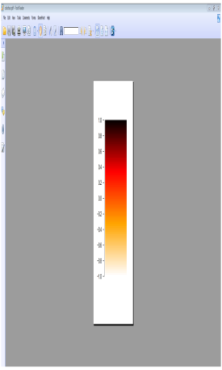


50-

0-


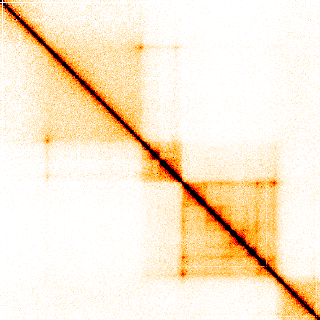

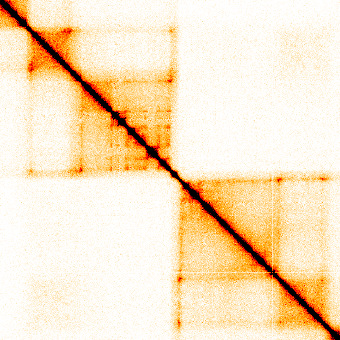

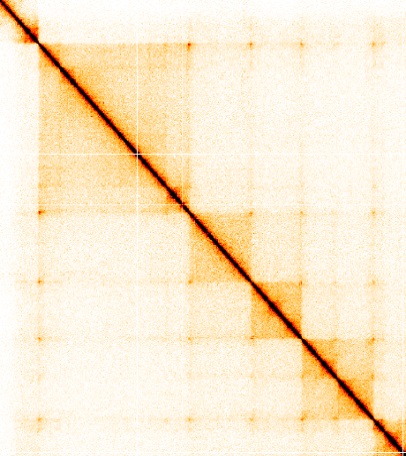


**Figure S1: Normalized contact maps generated by HiC-Pro at a 5kb resolution.** Example of chromatin loop structures observed at a 5Kb resolution using the IMR90_CCL186 data on chromosomes 3, 16 and 21.


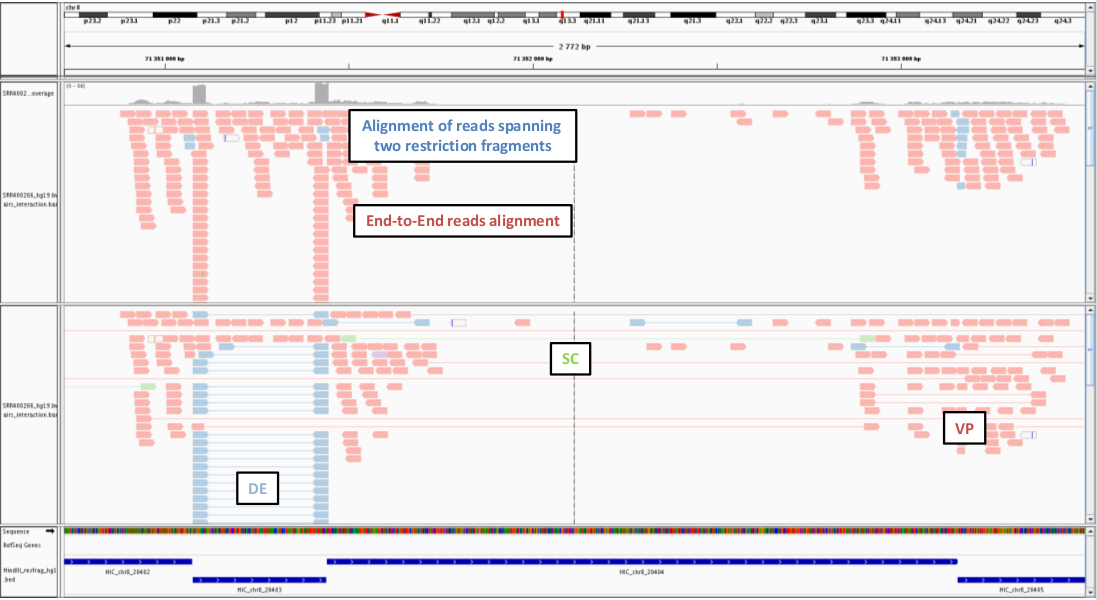


**Figure S2: IGV screenshot of BAM file after mapping and fragment reconstruction.** **Top panel.** The reads are colored according to the alignment procedure. Blue reads were trimmed before mapping, and flanked the restriction fragment borders. **Bottom panel.** Read pairs are colored according to their classification. Valid interactions are in red, dangling end in blue and self-circle ligation in green.


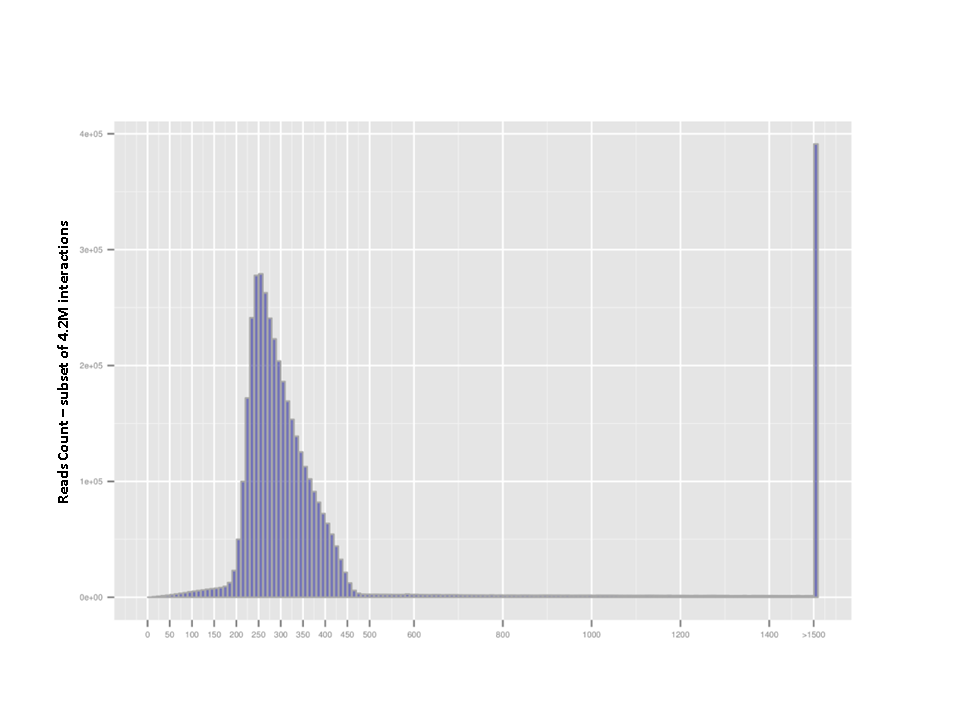


**Figure S3: Size distribution of Hi-C ligation products generated by HiC-Pro (IMR90).** Both reads are expected to map near a restriction site, and with a distance within the range of molecule size distribution after shearing. Fragments with a size outside the expected range can be discarded if specified in the HiC-Pro configuration file.

.
